# Reported infant feeding practices and contextual influences on breastfeeding: qualitative interviews with women registered to MomConnect in three South African provinces

**DOI:** 10.1186/s13006-020-00315-7

**Published:** 2020-09-14

**Authors:** Zara Trafford, Sara Jewett, Alison Swartz, Amnesty E. LeFevre, Peter J. Winch, Christopher J. Colvin, Peter Barron, Lesley Bamford

**Affiliations:** 1 Division of Social and Behavioural Sciences, School of Public Health and Family Medicine, University of Cape Town, Cape Town, South Africa; 2 School of Public Health, University of the Witwatersrand, Johannesburg, South Africa; 3 Department of International Health, Johns Hopkins Bloomberg School of Public Health, Baltimore, USA; 4 Division of Epidemiology and Biostatistics, School of Public Health and Family Medicine, University of Cape Town, Cape Town, South Africa; 5 Department of Public Health Sciences, University of Virginia, Charlottesville, USA; 6 Department of Epidemiology, School of Public Health, Brown University, Providence, USA; 7 National Department of Health, Pretoria, South Africa

**Keywords:** Breastfeeding, Infant feeding, Influences on decision-making, Behavioral determinants, mHealth, South Africa

## Abstract

**Background:**

Global guidelines recommend exclusive breastfeeding (EBF) for the first 6 months of life. South African EBF rates have steadily increased but still only average 32% for infants below 6 months of age. Malnutrition and developmental delays continue to contribute substantially to the morbidity and mortality of South African children. MomConnect, a national mHealth messaging system used to send infant and maternal health messages during and after pregnancy, has a specific focus on improving rates of breastfeeding and has achieved high rates of population coverage.

**Methods:**

For this qualitative study, we interviewed women who were registered to MomConnect to investigate their breastfeeding and other infant feeding practices, decision-making pre- and post-delivery, and the role of the health system, family members and the wider community in supporting or detracting from breastfeeding intentions. Data were collected from February–March 2018 in South Africa’s KwaZulu-Natal, Free State and Gauteng provinces. Framework analysis was conducted to identify common themes.

**Results:**

Most women interviewed had breastfed, including HIV-positive women. Even when women had delivered by caesarean section, they had usually been able to initiate breastfeeding a few hours after birth. Understandings of EBF varied in thoroughness and there was some confusion about the best way to cease breastfeeding. Most women felt well-equipped to make infant feeding decisions and to stick to their intentions, but returning to work or school sometimes prevented 6 months of EBF. Advice from the health system (both via clinics and MomConnect) was considered helpful and supportive in encouraging EBF to 6 months, although family influences could thwart these intentions, especially for younger women. Mothers reported a range of breastfeeding information sources that influenced their choices, including social media.

**Conclusions:**

Efforts to improve EBF rates must include consideration of the social and economic environment surrounding women. Interventions that focus only on improving women’s knowledge are valuable but insufficient on their own. Attention should also be paid to infant behaviors, and how these affect women’s breastfeeding choices. Finally, although there is strong local policy support for EBF, more rigorous implementation of these and other broader changes to create a more enabling structural environment ought to be prioritized.

## Background

The first thousand days of a child’s life influence their lifelong health and development trajectories, and nutrition and parental bonding are critical aspects of this stage of early childhood development. The World Health Organization (WHO) indicates that optimal feeding practices should include exclusive breastfeeding (EBF) for the first 6 months and continued breastfeeding for up to 2 years, a guideline which has been adopted by the South African government [1]. The bonding benefits of breastfeeding have also been established [2]. The most common feeding choices for South African infants, however, often do not align with this WHO standard [3, 4]. By 2016, 32% of infants under 6 months were being exclusively breastfed, an improvement on prior EBF rates but far from optimal [5]. Only 19% of children were breastfed between the ages of 12 and 23 months [5]. These sub-optimal feeding practices are a significant contributor to South Africa’s poor child health outcomes, with malnutrition still accounting for high rates of morbidity and mortality in the under-5 population [6].

**Fig. 1:**
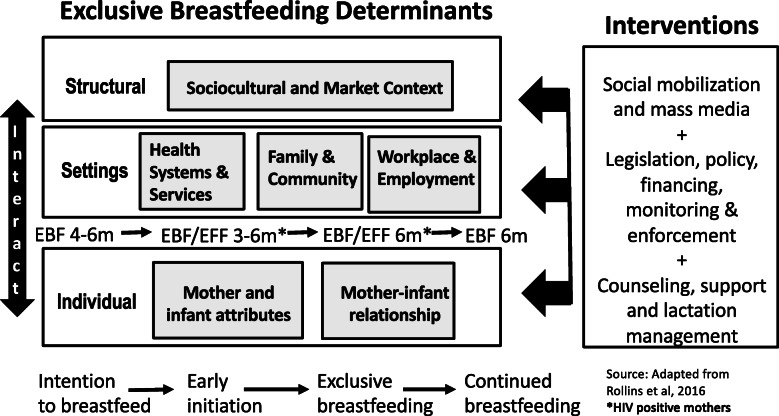
Breastfeeding Conceptual Framework for South Africa [8]

In order to identify appropriate interventions to promote breastfeeding, Rollins et al. [7] have proposed that breastfeeding determinants should be analyzed using a socioecological framework. The framework was further adapted by Nieuwoudt and colleagues [8] to account for intention during the antenatal period, as well as South Africa-specific feeding guideline changes over time (Fig. 1). Between 1980 and 2018 these guidelines shifted from EBF for 4–6 months for all women, to either EBF or exclusive formula feeding (EFF) for the first 3–6 months for women living with HIV, to either EBF or EFF for 6 months for women living with HIV, to EBF for all women for a full 6 months [8]. The framework emphasizes the importance of considering both *interpersonal* and *structural* contexts, in addition to the maternal attributes that have traditionally been the focus of breastfeeding behavioral change efforts, when investigating how feeding practices are explained or predicted.

Promotion of increased exclusive and continued breastfeeding is an important public health goal for South Africa. Accurate information is especially important in a high burden HIV context where guidelines for HIV-positive women initially encouraged infant formula feeding but have now evolved to reflect the recommendation that HIV-positive women on ART ought also to EBF for 6 months [8]. Despite strong policy support [9], challenges persist in the implementation of supportive regulations and optimal behavioral change has not yet been secured [4, 10, 11].

As one of a range of strategies to increase local EBF rates, a maternal mobile health (mHealth) program known as MomConnect was introduced nationally in 2014 to improve access to health information for pregnant and postpartum women. Women registered with MomConnect receive health information text messages via standard short message service (SMS) on their mobile phones and have access to a helpdesk which allows them to ask questions as well as register ‘compliments’ or ‘complaints’ about health services received [12]. Information on infant and maternal nutrition is the largest topic area, occupying 17% (24/144) of the total messages. MomConnect reaches more than 1 million pregnant and postpartum women annually, having reached 60% population coverage [13, 14]. This investment in pregnant and postpartum women’s access to information supports their improved knowledge and understanding and may result in improvement in infant feeding practices. However, when viewed within the framework of the socioecological model (Fig. 1), access to information is only one aspect of the widespread behavioral change needed. Improved individual knowledge must be supported by broader societal and structural changes in order to yield advances in EBF and continued breastfeeding rates.

The results presented in this paper are drawn from qualitative interviews conducted with women registered with MomConnect. The findings reported here explore two research questions: 1) among mothers registered with MomConnect, how were infants fed during their first 6 months of life? And 2) what were the key influences on these mothers’ infant feeding decision-making processes? Elsewhere, we present findings from the same study on user perceptions of MomConnect infant feeding messages (including content, timing, frequency and delivery modality) and generate recommendations for optimizing the program [15]. Using the adapted socioecological framework above (Fig. 1), this paper presents evidence toward a richer understanding of women’s practices around breastfeeding, key influences on their decision-making and what might facilitate or prevent the enactment of their breastfeeding intentions.

## Methods

### Setting

The results presented here are from the qualitative component of a multiple mixed methods study titled, “Linking Digital Health with Changes in Nutrition Outcomes: a formative research study”. Fieldwork was conducted in three South African provinces (Free State, Gauteng, and KwaZulu-Natal) selected with input from the National Department of Health because these three provinces have the poorest baseline child nutritional status [5]. Study sites included city centers and informal settlements in both urban and peri-urban areas.

### Sampling and recruitment

In each province we sampled from one metropolitan and one district municipality, which were purposely selected based on high registration rates to MomConnect. Women over the age of 18 with an expected due date of delivery between April and October 2017 were recruited using a series of SMS surveys sent out in English and the most common local language in each region – isiZulu (KwaZulu-Natal, Gauteng) or Sesotho (Free State). The surveys were distributed by Praekelt.org, a service partner also responsible for distributing MomConnect messages. The recruitment survey confirmed eligibility criteria, gathered respondents’ reported feeding practices (EBF, mixed feeding, no breastfeeding) and obtained initial consent to be contacted for an interview. The women who agreed to participate were contacted telephonically to arrange individual in-depth interviews (IDIs) or focus group discussions (FGDs).

A conscious effort was made to recruit women who reported different feeding practices to enable deeper exploration of infant feeding determinants. It proved impossible to recruit women into the final sample that were representative of distinct patterns of infant feeding for three reasons. First, the patterns of feeding were self-reported and not verified through observation. Second, the interviewers included breastfeeding women who consented to participate in the study but were not able to select women based only on their feeding patterns, as recruitment depended in part on participants’ availability for interview. Third, some women had patterns of feeding that did not fit within any of the standard definitions.

### Data collection

Data collection activities included IDIs and FGDs. Semi-structured interview schedules covered topics including infant feeding practices and experiences of and feedback about the content and impact of MomConnect infant feeding messages. The interviews also included conversations about the timing, delivery method and content of MomConnect messages; these data are reported elsewhere [15]. Semi-structured interview guides for individual and focus group activities were designed iteratively and collaboratively. An initial list of content areas and draft questions were prepared by the study designers who are mHealth and breastfeeding experts respectively. This list was then revised by the first author based on prior experience conducting interviews in South Africa. The guides were then refined during a two-day, in-person session with the research team and four research assistant interviewers experienced in conducting research in the specific research regions and with the relevant language skills. Once the interview guides were finalized the research assistants began conducting interviews and were advised weekly by the broader team during video call sessions.

The data collection teams consisted of 1–2 female fieldworkers with postgraduate training, qualitative research experience and appropriate disciplinary skills. All were working or studying at an academic institution at the time. None were affiliated with MomConnect. Prior to data collection, the fieldworkers were trained in a two-day in-person workshop. All fieldworkers were black African women, as were most of the participants interviewed. For each interview at least one fieldworker could converse fluently in the most common language (isiZulu in Gauteng and KwaZulu-Natal; Sesotho in the Free State) and conducted the interview either in English or in isiZulu or Sesotho, depending on the participant’s preference.

Data were collected from February to March 2018. At least one fieldworker in each team was fluent in English and the most common local language. All IDIs and FGDs were audio recorded. During the data collection the fieldworkers submitted written debriefs on each activity which were reviewed by the research team. Weekly check-in calls allowed the research team to make minor adjustments to the fieldworkers’ approach to obtain richer data.

### Data management

All files were stored in password-protected cloud storage shared only with co-investigators, the project manager and fieldworkers. Numbers were used as participant identifiers (e.g. FS IDI 01 for the first individual in-depth interview conducted in the Free State) and to name the files. Any direct quotes used here have been anonymized with these numbers.

### Data analysis

The initial data analysis took place through an iterative process led by the research team, who discussed the emerging findings with fieldworkers halfway through data collection. These findings were refined into themes through a full-day, in-person analysis workshop with the research team from the University of Cape Town following conclusion of the data collection.

We then used a framework analysis [16] to develop an initial codebook, based on the socioecological conceptual framework (see Fig. 1). The emergent codes were compared with the original inductively developed themes. The first author coded at least one interview from each province to “test” the codebook and the second author reviewed and added to these codes. The codebook was sent to the multilingual fieldworkers to review, with a focus on whether these themes had appeared in transcripts of other languages. Following agreement on the coding framework, the first and second authors used NVivo 12 to independently code the English transcripts. The fieldworkers then used the same codebook to analyze a further random sample of non-English transcripts and submitted additional indicative data to translate to English.

The first and second authors then worked together to produce an outline of findings and expanded on these independently. The broader research team provided input on drafts until consensus was reached [17]. The diversity of the authors in terms of gender, areas of expertise, age and personal experiences of infant feeding, facilitated rich discussions in the drafting and redrafting of the manuscript. The first and second authors are both women who work in public health and have postgraduate training in social science. The second author who is a breastfeeding specialist selected the chosen framework. Two of the other co-authors are breastfeeding and mHealth specialists respectively. A further two co-authors have worked or work in public sector health services in South Africa, were involved with the design and implementation of the MomConnect service, and have an interest in understanding and optimizing this program’s effects.

### Ethics

Ethical approval was obtained from the University of Cape Town Faculty of Health Sciences Human Research Ethics Committee. Department of Health approval in all three provinces was also obtained.

Informed consent was obtained from all participants prior to conducting data collection. Consent forms were available in English and the most common local language (isiZulu or Sesotho). Participants were given a copy which included contact details for the principal investigators and ethics committee. For FGD participants, we indicated that anonymity could not be guaranteed but that names would always be protected. Participants were interviewed in a language of their choice by fieldworkers unaffiliated with MomConnect to reduce bias. All transcripts were anonymized prior to analysis.

## Results

A total of 115 women participated in this study. The data collection activities comprised 67 IDIs and 12 FGDs (see Table 1).

**Table 1:** Data collection activities conducted

	**KwaZulu-Natal**			**Free State**			**Gauteng**		
	**Total**	**Metro**	**Non-Metro**	**Total**	**Metro**	**Non-Metro**	**Total**	**Metro**	**Non-Metro**
**IDIs**	**27**	14	13	**24**	15	9	**16**	10	6
**FGDs**	**4**	2	2	**4**	4	0	**4**	2	2

### Reported infant feeding practices

#### Breastfeeding

Some women were unable to breastfeed due to influences in their broader individual or social contexts, but at least some breastfeeding was reported by the majority of women interviewed across the three provinces, including mothers living with HIV. However, considerable variation emerged in practices regarding the timing of breastfeeding initiation, duration of breastfeeding and mixed feeding practices. Caesarean delivery, difficulties with latching and milk flow were the most common reasons for delayed initiation. Two mothers mentioned having diabetes as a reason for delaying breastfeeding initiation.

*[I breastfed] after 6 hours because I [had] an operation (C-section). They told me I had to [stay in] the same position* (GP IDI 6)

*I had to go the hospital every 2 days for them to assist [my baby] to latch* (FS IDI 17)

*I did not have milk for 3 days... the nurses fed my baby glucose water for 3 days* (KZN IDI 24)

Many women reported EBF, although they did not necessarily use the term “exclusive breastfeeding”. When probed, most correctly explained that EBF meant that they should not give their baby anything other than breastmilk, not even water, in the baby’s first few months of life.

*The baby needs [only] breastmilk for the first 6 months. Don’t give water, porridge or [any other food] you make* (KZN FGD 3)

There was sometimes confusion about the guidelines for exclusive breastfeeding. Some women reported having exclusively breastfed for the first 6 months but later in the same interview, reported giving their baby water before the recommended 6 months. Occasionally this was because they thought their baby was thirsty but more often, this was a once-off event for a specific reason, usually because of cultural beliefs or traditional practices. Even if such practices interrupted EBF from birth to 6 months, EBF or at least some breastfeeding were often adopted immediately afterward. These practices were not offered as a reason not to breastfeed at all.

Reasons for giving their baby water varied. Some women expressed a perception that the baby’s stomach needed to be cleansed after birth. For example, one woman gave her baby “starch water” (water with salt and sugar) as a stomach cleansing remedy. This common oral rehydration solution seems to have been conflated with a cleansing method. Another said she had given her baby glucose water to clean the baby’s tummy before taking it home from the hospital. There were also examples of visits to traditional healers for ritual stomach cleansing or other interventions.

*My baby had a mark on her face and cried all the time. I took her to a herbalist, he made small incisions on her wrists and... gave her medicine and also cleansed her [inside]*... *this really did help* (KZN FGD 4)

Infants were sometimes even prescribed water or herbal teas as a method for resolving breastfeeding issues. Visits to traditional healers did not emerge as a substantial pattern.

Overall, the women we interviewed seemed to be reporting that they were exclusively breastfeeding because it was their current or predominant practice, rather than accounting for consistency over the full six-month period. For example, one woman said she had tried breastfeeding, but her baby would not accept breastmilk and drank only water for a week. In desperation, this mother fed her baby infant formula and he was then able to breastfeed, so the formula was abandoned and EBF was adopted. In another case, a woman talked about giving her baby formula when he was not accepting breastmilk but when the baby threw up, she stopped giving him formula and returned to exclusive breastfeeding.

Beyond 6 months some women continued EBF, contrary to guidelines, but most reported complementary feeding practices. Many women said that they hoped to breastfeed for at least a year, with a minority citing an intention to continue until 18 or 24 months.

#### Infant formula use

Formula feeding was much less common than breastfeeding, although some practiced mixed feeding. Some who had exclusively breastfed until a certain point talked about having used formula as a “first food” to facilitate the transition from breastmilk.

#### Complementary feeding

Common first (complementary) foods included homemade soft porridge, mashed vegetables, and branded instant porridge and baby foods marketed for infants. Mothers sometimes mixed formula or other food with breastmilk to assist breastfeeding cessation. Some women complained they found it more difficult to transition their breastfed babies to complementary feeding.

*[EBF]*... *is giving us a problem because*. *.*. *when you start giving them food after 6 months, they don’t want it*... *I have a problem with this [baby], I fall asleep with her suckling* (GP FGD 4)

*I have started to introduce other foods, but she is very reluctant to eat them. She loves her breastmilk way too much* (FS IDI 15)

#### Bottle use

Both breastfeeding and infant formula feeding women reported using bottles, but the need to clean and store bottles thoroughly to prevent illness was also considered an explicit deterrent to bottle-feeding and cited as a motivator for breastfeeding.

*Breastmilk is easy because when your baby cries, you take out your breast, wipe it and feed your baby*. *.*. *this prevents illness* (KZN FGD 2)

*Breastmilk*... *is not a hassle because it has water and all the nutritious value, you don’t have to carry anything extra. It is “all-in-one”* (FS FGD 4)

### Influences on breastfeeding

#### Mother-specific influences

##### Mothers’ knowledge and beliefs

Knowledge about the health benefits of breastfeeding seemed relatively good, even among those who were unable or chose not to breastfeed. Many believed that non-breastfed babies got sick more often or were under-nourished, and some made connections with later childhood development. Along with generic references to the healthiness of breastmilk, some women reported using it as “medicine” (e.g. putting breastmilk in a baby’s nose to clear congestion). Breastmilk was generally believed to be superior to formula, with some women expressing concerns about harmful chemicals in formula. Some breastfeeding women were gently critical of non-breastfeeding mothers.

*This [breastfed baby] has never been sick. Breastmilk is number one! (FS IDI 24)*

*Some babies are slow learners at school because they were not breastfed (FS FGD 4)*

Mothers also mentioned bonding, cost savings, and simplicity, when compared to preparing formula and bottles, as perceived benefits of breastfeeding.

*The first day felt weird because I wasn’t used to it but as time went on, [we] established a strong bond*... *I could breastfeed forever* (FS IDI 23)

Affordability was most often raised by unemployed women, with one mother exclaiming “... it’s much cheaper... say goodbye to R500 per month [for formula]!” (GP IDI 8).

Women were less certain about *why* it was important to breastfeed for 6 months, who should do so, and when and how best to cease breastfeeding. For example, one mother took a health worker’s (HW) recommendation to EBF for 6 months very literally and stopped breastfeeding altogether at exactly 6 months. Another woman believed that the recommendation to EBF for 6 months was only relevant if “the baby doesn’t give you problems” (GP IDI 10). These women seemed confused about exactly what would happen if they did not breastfeed and whom to trust most when unsure what to do. Finally, mothers described proactively seeking feeding information from the internet. One even mentioned learning about the nutritional sufficiency of breastfeeding from information on a formula tin.

##### Problems with breastfeeding

Some women talked about having had difficulties with breastfeeding such as pain from over-full breasts, difficulty latching, inverted nipples or difficulty bonding because of postnatal depression. Among our sample these concerns were generally overcome, usually with assistance or advice from HWs. Issues that prevented or stopped breastfeeding included sores on the breast or pus in the milk and a belief that their milk was “insufficient”. Others cited caring for other children or competing demands as barriers to EBF and continued breastfeeding.

*I mixed at 3 days after birth*... *[It was] more convenient because [I have another] small child* (KZN IDI 6)

*I had to look after the baby, clean and cook. I was [too] tired [to breastfeed]* (FS IDI 18)

##### Mothers’ HIV status

Both HIV-positive and HIV-negative women seemed to know the importance of EBF for preventing mother-to-child transmission of HIV. Some women specifically referred to their HIV status as the primary reason for breastfeeding. Although others said they still struggled to believe the recommendation, support for EBF was reinforced when they observed that their infants had not seroconverted.

*The first time, I was scared [to BF]*.. *because I am HIV-positive*... *[but] I gave my baby breastmilk [and] he tested negative* (KZN IDI 12)

Most HIV-positive women believed that mixed feeding was dangerous and that they should choose either EBF or EFF. Some women specifically noted the importance of proper adherence to antiretroviral treatment while breastfeeding.

Disclosure to family and friends helped HIV-positive women talk about and obtain support for their decision to exclusive breastfeeding. Some felt pressured to keep their babies with them at all times in case somebody mistakenly disrupted exclusive breastfeeding.

#### Infant influences

Mothers and other caregivers often followed their infant’s cues, for example, crying, sleeping habits changing, eating too much or too little which could either prompt or discourage breastfeeding. A key issue was that mothers and their relatives usually assumed that hunger was the main reason for a baby crying. If the baby was crying a lot, the intention to EBF could be thwarted. Sometimes mothers, even among those who had chosen to EBF, did not seem to believe that breastmilk alone was enough to satisfy an infant, describing breastmilk as “just liquid” (FS ISI 17) or “water”. One mother asked in confusion, “I don’t understand it, because a baby is supposed to eat! Or do small babies not get hungry?” (FS IDI 14). These ideas were closely linked to beliefs about insufficient milk.

... *the baby would cry the entire night*... *I realized that*... *I [was] not making enough milk and decided to buy formula* (GP IDI 8)

Observations of an infant’s physical appearance were regularly used to reinforce feeding choices, especially among women who had breastfed. Baby’s bodies were cited as evidence of the healthiness of breastfeeding, with some mothers urging the interviewer to look at their “big and strong” breastfed baby. Conversely, if a baby was perceived to be malnourished, this was a cue to begin mixed or complementary feeding before 6 months of age.

#### Influences in the healthcare setting

##### Health worker advice

HWs’ advice was repeatedly cited as a key source of breastfeeding support. Advice received at clinics was generally considered trustworthy and more accurate than information from other sources. HWs did not seem to be recommending infant formula use unless there was a profound issue with breastfeeding. Even then, many women reported that HWs encouraged them to keep trying to breastfeed. One woman described the six-month EBF recommendation as the nurses’ “favorite song” (FS IDI 6). The HWs’ approach to encouraging EBF varied but could be quite forceful.

*At the clinics, [HWs] can be pressurizing. They give you that eye, like no-no to formulas*... *but [ultimately] it’s up to you. You don’t have to feel forced* (KZN IDI 25)

HWs were noted as the primary source of HIV-positive mothers’ strong knowledge of the interplay between their status and breastfeeding. Women sometimes used this information strategically to convince older relatives of the importance of breastfeeding in the context of high rates of infectious and chronic illness, although this was not always successful.

... *I explained to [my grandmother] that these days, babies do not need water if they are breastfeeding, and that things have changed. I*... *explained to her that we are trying to avoid many sicknesses by breastfeeding the child* (FS IDI 16)

Responses to HW advice varied depending on women’s observations of HW behaviors. Those who mistreated people in their community were discounted: “… people… do not follow the nurses’ instructions because they say that nurses have [an] attitude…” (FS IDI 14). Some mothers noted that HWs did not EBF when feeding their own infants, which undermined their advice. There were also reports of conflicting advice given by HWs, which decreased overall faith in clinic recommendations and could thwart EBF intentions.

*I was*... *afraid to give [my baby] water. I*... *asked the nurse what to do. [She] said, ‘Ok give her a little bit of water, just a little bit’* ... *You just don’t know [what to do!]* (GP FGD 4)

##### Caesarean sections

The relationship between caesarean section (C-section) and breastfeeding varied. Two women described difficult or traumatic C-section experiences, which had delayed initiation or made them feel bitter about breastfeeding. In contrast, others explained that they were given their babies to breastfeed very soon after their C-sections. Women were often unclear about what their infants were fed when they were separated, a period which ranged from several hours to a week.

*[I breastfed] a day after [birth]*. *.*. *I’m sure they gave him something [to eat]*. *.*. *because they took him from me. I [delivered] him by Caesar*... (GP IDI 3)

##### Other breastfeeding promotion

MomConnect messages (often used in conjunction with HW advice) were noted as a very valuable source of information that supported women’s knowledge and decision-making. One Gauteng woman described how MomConnect had stopped her from discontinuing breastfeeding. For some, MomConnect was the first source of the six-month EBF recommendation. Mothers specifically highlighted MomConnect recommendations for the transition to complementary feeding, which were more specific and precise than clinic advice.

Women found MomConnect empowering and had sometimes used the messages to teach their peers and family about pregnancy and infant care, although this was not always successful. Some used the messages to resist pressure not to breastfeed by “proving” its importance.

*My mother [said]*.*.*. *‘every child needs water and is supposed to be given her first drink of water when she arrives at home to cleanse her bellybutton’. I told her that breastmilk has the same ‘water’ for cleansing* (FS IDI 8)

There was also evidence of some health facilities following baby-friendly policies and using breastfeeding promotional materials. Interviewees sometimes referred to these materials, but they were not considered the main motivator of breastfeeding.

#### Family influence

Mothers consulted with their family but usually characterized infant feeding decisions as their own. Intimate partners or the child’s father were sometimes consulted but did not play a central role. Most felt able to enact their decisions. However, influences from family or the need to return to work or school could be powerful deciding factors when it came to the enactment of breastfeeding intentions. Those who had not breastfed tended to be younger, financially dependent on family, involved in shared caregiving or reliant on older female relatives’ experiences as a model for their choices. These women had even less decision-making power if they were going to leave their baby with their family while they went away for work or schooling.

*I was going to breastfeed for the first 6 months*... *when I was pregnant, I said to my mom I am going to buy a breast pump*... *She said, ‘No, you are not going to be here [because you’re away studying] so we must just start with formula while you are here so that the baby does not cry [when you’re away]’.* (GP IDI 8)

In contrast, some said that their mothers had encouraged or even pressured them to breastfeed but that EBF was not generally preferred. Instead, complementary feeding was recommended.

*[Older women] believe when a baby cries it means they are hungry. They don’t believe in EBF but believe in [complementary feeding and] breastfeeding for about 2 years.* (FS IDI 22)

#### Community influences

Many women felt uncomfortable “taking their breasts out” in public, although most persevered. One woman laughed at the idea of breastfeeding her baby while sitting at a restaurant with her friends. Women in two provinces also described an undesirable association between poverty and breastfeeding that could act as a deterrent.

*When you breastfeed, people think you’re poor*.*.*. *higher class people don’t breastfeed, they can afford formula* (KZN FGD 4)

A small number said that their decision to formula feed was motivated by aesthetic concerns about the appearance of their breasts. A more prominent concern was women’s confidence, or lack of confidence, to feed in public. Mothers feared judgment from others. Discomfort with public breastfeeding also had a gendered dimension. In one FGD, participants explained that other women were less accepting than men of public breastfeeding. In contrast, another young woman described her discomfort about breastfeeding in the home environment due to the presence of male relatives, an idea imposed by her mother.

#### Work or school influences

Planned or forced returns to work or school were the main reason for selecting formula or mixed feeding before 6 months, as well as influencing when women began complementary feeding. Conversely, unemployment facilitated continued breastfeeding, partly because unemployed women could spend more time with their baby and partly because breastfeeding was more affordable. One woman even left her job so that she could breastfeed. There was minimal evidence of maternity leave being available in this sample of women but where noted it was clear that maternity leave facilitated breastfeeding only until the end of the leave period.

## Discussion

While South Africa has seen an improvement in breastfeeding rates, and initiation is common, EBF and continued breastfeeding rates are still below recommended global targets and continue to be a public health problem. As per the adapted socioecological model (see Fig. 1), interventions at multiple levels are required if widespread and profound behavioral change is to occur. These changes include factors at the individual (mother's) level such as knowledge, but also expand to include factors at the community, health system and broader societal levels. Below we discuss our findings within this framework and offer suggestions based on existing local and global evidence.

### Infant feeding practices

Women in this study reported relatively high rates of EBF and continued breastfeeding, which is consistent with a recently observed trend toward increased breastfeeding in South Africa [4–6, 10, 18]. As other studies have found, this trend was also true for HIV-positive mothers, some of whom explained that their HIV status had motivated them to choose breastfeeding over alternative feeding methods [19].

However, the variable and incomplete understanding of EBF identified among some women in this study reinforces the observation that the way women define EBF *qualitatively* does not necessarily align with the standard WHO definition. Women’s reports of their actual practices and understanding of the concept of exclusivity diverged from the more rigid WHO guideline definition, and they also seemed less sure about *why* they should EBF for 6 months. Some researchers already measure breastfeeding practices along a continuum [20], which is more aligned with community-led definitions of exclusive breastfeeding. It is likely practicing EBF for a longer period could be encouraged if EBF is considered a practice that mothers can return to if they have lapsed, rather than applying an inflexible measure which no longer considers an infant exclusively breastfed if they are fed water once.

Among our sample, infant formula was usually used for practical reasons like a return to work or school, as in other recent local studies [19, 21]. Complementary feeding included mention of both homemade and commercial products. The preponderance of the latter, advertised as nutritionally valuable, is of growing concern due to high sugar content [22, 23]. Women were sometimes confused about when to introduce complementary foods as a result of their uncertainty about health messages or conflicting messages from families. Such confusion can undermine self-efficacy to breastfeed, which is linked to successful exclusive breastfeeding [24].

### Individual-level influences

Among mothers, confidence in the superiority of breastmilk over formula seems to be gaining traction [18, 19]. However, beliefs about milk insufficiency persisted in some contexts, sometimes even among women who reported exclusive breastfeeding. Our observations of mixed feeding or early breastfeeding cessation being linked to nutritional concerns in the first 6 months also echoes the global literature indicating that fears about breastmilk insufficiency are a key barrier to exclusive breastfeeding [25]. Despite promising efforts by government and civil society to reinforce the sufficiency of breastmilk during the first 6 months [12, 26], such beliefs persist.

Findings suggest that HIV-positive mothers could benefit from additional specialized interventions. WHO guidelines for HIV-exposed infants were revised in 2016 to indicate that there is a low risk of transmission during mixed feeding if the mother is virally suppressed [1]. However, mothers remained convinced about the dangers of mixed feeding and this is consistent with similar evidence from the rest of the country about the persistence of earlier guidelines [18, 19, 27]. Our findings also reinforce the importance of helping mothers disclose their status to their families in the context of ongoing HIV stigma [28], as those who had been able to disclose felt they were more supported in their breastfeeding intentions.

The health information consumption behavior of South African mothers remains understudied in public health, but global trends suggest a movement towards social media as a key source [29]. Nationally, the penetration of mobile devices is high and compared to some other parts of the global South, women’s access to these devices is also relatively high [12]. Some women in this study did describe using social media sources to find additional information about messages or advice they had been given, but this was not systematically probed. This is a potentially rich area for additional or complementary interventions.

With some exceptions [30, 31], the influence of an infant’s behavior and growth on feeding choices are often overlooked in quantitative studies. In contrast, many qualitative studies, including this one, indicate that feeding choices are often made based on infant cues [24, 32]. Our study underlines the critical importance of supporting caregivers to appropriately interpret infant cues (especially crying or sleeping), which often resulted in the cessation of breastfeeding or early infant formula introduction. This study also speaks to the importance of observing and considering infants, as well as mothers, when feeding issues are reported, as efforts to influence feeding choices are often more focused on addressing a mother’s individual behavior.

### Healthcare influences

Caesarean delivery did not emerge as a significant barrier to EBF to 6 months. Although women who experienced a C-section delivery reported slightly delayed breastfeeding initiation, they did not link this experience with prevention of exclusive breastfeeding. A similar pattern was reported in recent meta-analyses and systematic reviews, which found associations with delayed initiation but not with EBF to 6 months, as long as women were adequately supported to breastfeed following C-section [7, 33]. Encouragingly, the women in our study often cited the advice of HWs as a positive and supportive influence on breastfeeding. In previous local research, advice has been described as more inconsistent [34, 35]. The way HWs acted toward women was also important, as observations of HW behavior sometimes mattered more than their instructions. This observation is important given the findings of a recent study of HWs that found that few were able to EBF their own infants [10], a situation also noted by one of the women in our study. Nevertheless, in cases where women received family advice that undermined their decision to EBF, they generally trusted HW advice and used it to defend their decision. The importance of HWs in shaping BF intentions and practice has been well-established locally and globally. Their ongoing influence reinforces calls for ongoing HW refresher training, as well as ensuring consistency in recommendations and consensus among HWs involved in ante- or postnatal care [10, 36].

The overall healthcare environment also supported or inhibited breastfeeding. The use of health promotion messaging was considered supportive but insufficient on its own to encourage widespread changes. Prior stand-alone efforts, like informative videos [37] or once-off counselling [38], have not yielded convincing results. In contrast, interventions that have applied health system-level reforms such as providing integrated care for mothers and infants have recorded notable EBF improvements [39]. Similarly, integrated approaches may be beneficial in continuing to turn the tide toward improved EBF rates.

Interventions that extend healthcare and interpersonal support *beyond* health facilities have also shown promise in South Africa and other LMICs [19, 40, 41]. MomConnect is one component of a national strategy to increase EBF rates and support new mothers. It buttresses broader health system efforts to promote these messages and offers a rare opportunity to reach most of the relevant population with evidence-based infant and maternal health information. Our qualitative findings reinforce an earlier qualitative evaluation of MomConnect that found improved breastfeeding self-efficacy among those who had been exposed to messages promoting breastfeeding [42]. Our study also illustrated how women use the information to “teach” their peers or strategically gain support for their feeding choices.

### Influences in the broader social environment

Most feeding practices happen outside health facilities. The role that family members play in either supporting or undermining feeding decisions [4] was reiterated by women in our study. Strong kinship networks and cooperative caregiving practices in the African context make negotiation with family members an important skillset, especially for young or first-time mothers [43].

Our study is not the first in South Africa to identify discomfort about breastfeeding in public as a potential barrier [44, 45], albeit at low levels. The sexualization of women’s breasts contributes to these fears. Concerns about breastfeeding in public may be partly linked to individual self-perception but these ideas may more accurately be framed as a reflection of internalized social norms about what is or is not acceptable in public. Whether real or perceived, the fear of drawing attention or judgement around breastfeeding is known to affect breastfeeding self-efficacy. Discomfort under the male gaze is cited in the global literature [46] and was observed in this study too. Interestingly, women in this study specifically mentioned other *women’s* negative judgments as a deterrent to breastfeeding in public. Changing attitudes more broadly will require intersectoral collaboration and interventions, as well as public campaigns to shift popular perceptions.

A novel finding in this study was the practice of a minority of mothers choosing to breastfeed rather than returning to school or work. Generally, work and school responsibilities undermined breastfeeding among women in this population, just as they do globally [7]. Inadequate labor policies and unsupportive environments are known barriers to breastfeeding. Of the minority of participants who were employed at the time of interview, only one talked about having been granted maternity leave. Attention to the work environment is increasing [9]. However, given the high proportion of young women (aged 15–19) giving birth in South Africa (71 births per 1000) [5], and younger women's reported reliance on family for childcare, the school environment should also be considered an important space for breastfeeding interventions.

### Limitations

Many of the women in our sample were unemployed. Based on existing evidence about the relationship between lower rates of employment and higher rates of breastfeeding [7, 8, 47, 48], these mothers were more likely to be both breastfeeding and available for daytime interviews. Participants were responding to recruitment messages that specifically inquired about breastfeeding practices and were also extensively questioned about breastfeeding and related practices during interviews, which may have resulted in a degree of social desirability bias. However, as is often the case with qualitative research, sampling was aimed at gaining deep, rich accounts of women’s feeding practices and decision-making influences rather than obtaining a representative sample. In order to gather varied experiences, women were purposively sampled from more than one province and both metropolitan and district municipalities within each province.

## Conclusions

Infant feeding decision-making and behaviors must be understood within an ecological context that extends beyond the traditional mother-infant dyad [3, 7, 18]. HWs and health-related channels like MomConnect are considered trusted sources for accessing accurate information. These sources are extremely helpful in shaping intention and supporting breastfeeding initiation but must be harmonized for best effect. The growth of virtual spaces such as social media present important new channels for future research or complementary interventions related to breastfeeding promotion, which could build on the gains made so far.

As well as a focus on improving informed support for mothers within the health system, efforts ought also to be directed at society at large. MomConnect is a powerful tool that can be delivered at scale to great effect, but its ability to address structural and behavioral barriers is limited. Our findings confirm that improving maternal knowledge and attitudes towards breastfeeding is valuable but insufficient as a standalone approach to widespread shifts in practice, particularly for young and working mothers. Women need more enabling environments, both structurally and in terms of norms and expectations, in order to apply and benefit from information.

## Data Availability

The primary data obtained through this component of the study was in the form of interview audio recordings and transcripts. The dataset generated and analyzed during the current study is not publicly available. There are important ethical and confidentiality reasons why in-depth interview data cannot be made open access, which would violate the terms of our ethical approval. We do not plan to provide direct access to the primary research data for these reasons, although additional data in the form of anonymized supporting quotes can be provided by the first author on reasonable request.

## Abbreviations

EBF: Exclusive breastfeeding
EFF: Exclusive (infant) formula feeding
FGD: Focus group discussion
FS: Free State (South African province)
GP: Gauteng Province (South African province)
HIV: Human immunodeficiency virus
HW: Health worker
IDI: (individual) in-depth interview
KZN: KwaZulu-Natal (South African province)
PMTCT: Prevention of mother to child transmission (of HIV)
WHO: World Health Organization
